# Retraction: The nuclear export of TR3 mediated gambogic acid-induced apoptosis in cervical cancer cells through mitochondrial dysfunction

**DOI:** 10.1039/d1ra90028e

**Published:** 2021-01-21

**Authors:** Laura Fisher

**Affiliations:** Royal Society of Chemistry Thomas Graham House, Science Park, Milton Road Cambridge CB4 0WF UK advances-rsc@rsc.org

## Abstract

Retraction of ‘The nuclear export of TR3 mediated gambogic acid-induced apoptosis in cervical cancer cells through mitochondrial dysfunction’ by Chunhong Zhang *et al.*, *RSC Adv.*, 2019, **9**, 11855–11864, DOI: 10.1039/C8RA10542A.

The Royal Society of Chemistry hereby wholly retracts this *RSC Advances* article due to concerns with the reliability of the data. The images in the article, and the raw data provided by the authors, were screened by an image integrity expert. All the western blot bands are uniform in shape, the panels are over-contrasted, have no background and do not look genuine. The authors have not been able to provide raw, unprocessed images to verify the validity of the published western blot images.

The authors contacted the Editor asking to retract the article as they found that the published data was not accurate through follow-up studies.

Given the significance of the concerns about the validity of the data in the article, the findings presented in this paper are not reliable.

The authors did not respond to any correspondence regarding the wording of the retraction notice.

Signed: Laura Fisher, Executive Editor, *RSC Advances*.

Date: 7^th^ January 2021.

## Supplementary Material

